# Estimating the Burden of Pneumococcal Pneumonia among Adults: A Systematic Review and Meta-Analysis of Diagnostic Techniques

**DOI:** 10.1371/journal.pone.0060273

**Published:** 2013-04-02

**Authors:** Maria A. Said, Hope L. Johnson, Bareng A. S. Nonyane, Maria Deloria-Knoll, Katherine L. O′Brien, for the AGEDD Adult Pneumococcal Burden Study Team

**Affiliations:** 1 Department of Medicine, Division of Infectious Diseases, Johns Hopkins School of Medicine, Baltimore, Maryland, United States of America; 2 International Vaccine Access Center, Johns Hopkins Bloomberg School of Public Health; University of Otago, New Zealand

## Abstract

**Background:**

Pneumococcal pneumonia causes significant morbidity and mortality among adults. Given limitations of diagnostic tests for non-bacteremic pneumococcal pneumonia, most studies report the incidence of bacteremic or invasive pneumococcal disease (IPD), and thus, grossly underestimate the pneumococcal pneumonia burden. We aimed to develop a conceptual and quantitative strategy to estimate the non-bacteremic disease burden among adults with community-acquired pneumonia (CAP) using systematic study methods and the availability of a urine antigen assay.

**Methods and Findings:**

We performed a systematic literature review of studies providing information on the relative yield of various diagnostic assays (BinaxNOW® *S. pneumoniae* urine antigen test (UAT) with blood and/or sputum culture) in diagnosing pneumococcal pneumonia. We estimated the proportion of pneumococcal pneumonia that is bacteremic, the proportion of CAP attributable to pneumococcus, and the additional contribution of the Binax UAT beyond conventional diagnostic techniques, using random effects meta-analytic methods and bootstrapping. We included 35 studies in the analysis, predominantly from developed countries. The estimated proportion of pneumococcal pneumonia that is bacteremic was 24.8% (95% CI: 21.3%, 28.9%). The estimated proportion of CAP attributable to pneumococcus was 27.3% (95% CI: 23.9%, 31.1%). The Binax UAT diagnosed an additional 11.4% (95% CI: 9.6, 13.6%) of CAP beyond conventional techniques. We were limited by the fact that not all patients underwent all diagnostic tests and by the sensitivity and specificity of the diagnostic tests themselves. We address these resulting biases and provide a range of plausible values in order to estimate the burden of pneumococcal pneumonia among adults.

**Conclusions:**

Estimating the adult burden of pneumococcal disease from bacteremic pneumococcal pneumonia data alone significantly underestimates the true burden of disease in adults. For every case of bacteremic pneumococcal pneumonia, we estimate that there are at least 3 additional cases of non-bacteremic pneumococcal pneumonia.

## Introduction

Community-acquired pneumonia (CAP) is a significant cause of morbidity and mortality among adults worldwide, of which a significant proportion is believed to be caused by *Streptococcus pneumoniae* (pneumococcus). The World Health Organization (WHO) estimated in 2005 that among all age groups, pneumococcal disease caused an estimated annual 1.6 million deaths [1]. Although the global pneumococcal disease burden among children is well understood, with an estimated 13.9 million cases of pneumococcal pneumonia occurring among children <5 years of age in 2000 [2], [3], the pneumococcal pneumonia burden among adults is not well characterized, impeding policy formulation for prevention and treatment.

In the pre-antibiotic era, when organisms were identified by culture and mouse inoculation, 95% of lobar pneumonia cases [4] were attributed to pneumococcus. *S. pneumoniae* is still thought to be the most common etiologic agent of CAP [5], [6], [7], [8] but is now identified in a much lower proportion of patients than historically.This change is thought due to a decrease in microbiological testing and the use of empiric antibiotics prior to testing, which has increased the proportion of cases with unknown etiology [9]. Many of these cases are believed to be attributable to pneumococcus [10].

**Figure 1:**
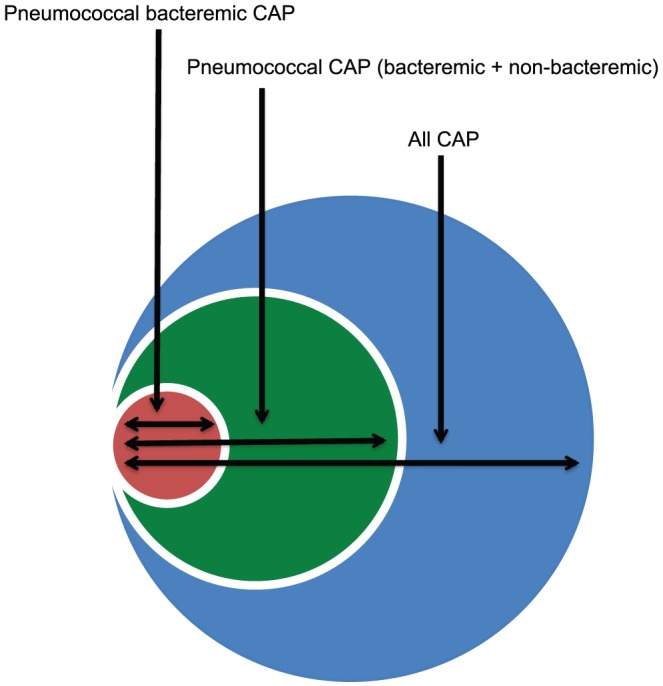
The relationships between CAP^1^, non-bacteremic pneumococcal pneumonia, and bacteremic pneumococcal pneumonia.

Our objective was to develop a conceptual and quantitative strategy to estimate the burden of non-bacteremic pneumococcal pneumonia among adults by assessing the yield of blood cultures, sputum cultures, and the Binax UAT to establish the proportion of pneumococcal pneumonia that is bacteremic and the proportion of CAP attributable to pneumococcus (Figure 1) (See Appendix S1: Said, MA, et al. Estimating the burden of pneumococcal pneumonia among adults: the conceptual framework). Here, we present estimates for these values, achieved through a systematic review and meta-analysis of studies that report results of the Binax UAT as well as blood culture and/or sputum culture. We also present an estimate of the additional contribution of the Binax UAT to the conventional diagnostic techniques of blood and sputum culture.

## Methods

### Search Strategy

A conceptual framework and modeling strategy was first developed (see Appendix S1). The model was used to guide this systematic review and meta-analysis, which was prepared in accordance with guidelines for meta-analyses of observational studies [11] as well as the PRISMA statement [12]. A previously existing review protocol for this particular study was not known to exist. We performed three separate literature searches in Medline, Embase, CINAHL, Global Health, and ISI Web of Knowledge without date restrictions to identify papers reporting the diagnostic yield of the BinaxNOW® *S. pneumoniae* urine antigen test (UAT) as well as blood culture and/or sputum culture in cases of CAP among adults and that would allow for a comparison between the Binax UAT and at least one of the other two tests. The literature search was performed by one author (MS) in May–June 2010 with the assistance of a medical librarian. Titles and abstracts were screened for potentially relevant citations and the full-text was retrieved for studies reporting the diagnostic yield of the BinaxNOW® *S. pneumoniae* urine antigen test (UAT) as well as blood culture and/or sputum culture in cases of CAP among adults. The first search aimed to identify papers that reported the usefulness of the Binax UAT in diagnosing pneumococcal pneumonia; in Pubmed, the following search terms were used: (("binax"[all fields] OR "binaxnow" OR "urinary antigen test")) AND ("Streptococcus pneumoniae"[Mesh] OR "Pneumococcal Infections"[Mesh] OR "streptococcus pneumoniae"[all fields] OR "pneumococcal"[all fields] OR "diplococcus pneumoniae"[all fields] OR "pneumococcus"[all fields] OR "pneumococci"[all fields] OR "S. pneumoniae"[all fields] OR "pneumococcal infection"). The second search aimed to identify papers that reported the usefulness of the Binax urinary antigen test in all cases of pneumonia; search terms used were: (("Pneumonia"[Mesh] OR "pneumonia")) AND ("binax" OR "binaxnow" OR "urine antigen" OR "urinary antigen"). The third search broadened the terms used to identify studies using the Binax UAT by including the search terms "streptococcus pneumoniae antigen" OR "urinary pneumococcal antigen". References lists of obtained papers as well as suggestions from other authors were used to identify additional studies.

**Figure 2:**
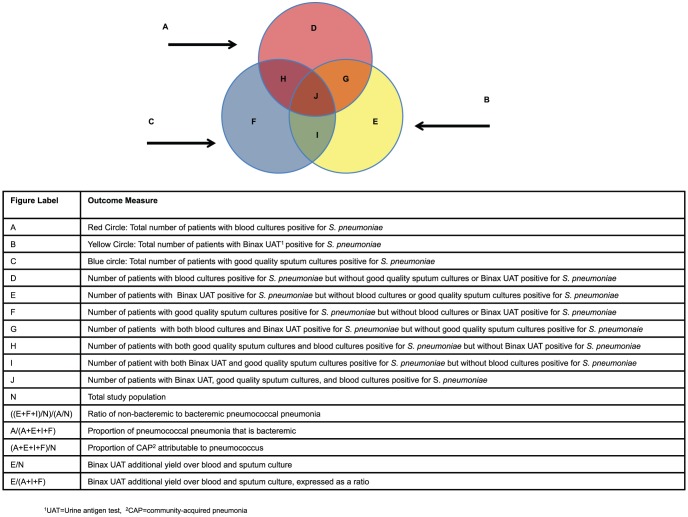
The relationship in diagnostic test yield of blood culture, sputum culture, and the Binax UAT^2^.

A study was eligible for inclusion if it reported primary data from human studies of radiologically confirmed adult pneumonia cases. We considered all studies that reported results of the Binax UAT as well as blood culture and/or sputum culture. We excluded studies that looked at particular patient populations that were not representative of the general adult CAP population; for example, a study conducted among patients with malignancy. We chose not to exclude a single paper in which all patients were infected with HIV, as we were particularly interested in how HIV infection might affect the outcome measures. Studies about cases already identified as pneumococcal were excluded in order to reduce bias. We included studies utilizing the Binax UAT on both concentrated and non-concentrated urine. Sputum culture results were accepted only if sputum quality criteria were used. All foreign language articles were translated, either through electronic or official translation services. Study population characteristics, case ascertainment and diagnostic methods, and relevant outcome data were abstracted from studies after full-text screening. We also attempted to contact the authors of potentially relevant studies to obtain additional information, including the number of patients undergoing each diagnostic test, the number of positive test results, and the overlap in positive results among the three methods for detection (Figure 2).

Pneumococcal pneumonia was defined as radiographic pneumonia in addition to at least one positive laboratory test: a blood culture or sputum culture positive for pneumococcus or a positive Binax UAT. Bacteremic pneumococcal pneumonia was defined as having a blood culture positive for pneumococcus, and non-bacteremic was defined as having a sputum culture positive for pneumococcus and/or a positive Binax UAT without a blood culture positive for pneumococcus.

### Study Selection

Of the 488 articles originally identified, 140 were chosen for full text screening. Of these, 75 were identified to have potentially useful information on comparative diagnostic yield of the tests; authors were contacted to provide additional information and clarification. When a subset of the study population contained a population of CAP patients thought appropriate for inclusion, (i.e. a study of patients with lower respiratory tract infections among whom some were identified as having radiologically confirmed CAP), the author was asked to submit data on the desired subset. When an abstract without a published paper was identified that was thought to represent a study that might include the needed data, the author was also asked for additional data. Authors known to have worked or be working on a study that was thought to be suitable but had not yet been published were also contacted. Contact was conducted over email; if no email address for a contacting author was identifiable, a letter was sent by mail. Several publications reported results from the same cohort of CAP patients and were classified as one study. Contact was made with a representative from 49 of the 75 papers; a completed abstraction form was returned for 33 study cohorts, not of all which were found to meet criteria to be included in the study. Six of the studies included in the final analysis were included based on only the information provided in the published paper, as we were unable to communicate with the author. Thirty-five studies were ultimately included in the analysis.

### Data extraction

Extracted data included the country in which the study was conducted, the time period over which the study took place, the language in which the paper was published, the nature of the study population, the presence or absence of a chest x-ray requirement in the diagnosis of pneumonia, the presence or absence of criteria by which to judge sputum quality, the mean age of participants, the percent of participants that were hospitalized, the percent of patients in an ICU, the mean PSI score and the distribution of participants among the five PSI classes, the percent of participants vaccinated against pneumococcus, the percent of patients with HIV, the percent of patients who came from a nursing home, the percent of patients who received antibiotics before diagnostic testing, the numbers of patients who underwent each diagnostic test, whether concentrated or unconcentrated urine was used to perform the Binax UAT, the numbers of study participants with positive blood cultures, sputum cultures, and the Binax UAT and the overlap in positivity of these test results, the numbers of participants with positive test results for each of the diagnostic tests studied stratified by whether they had received antibiotics prior to diagnostic testing, and the number of positive test results for each of the diagnostic tests studied stratified by the Pneumonia Severity Index (PSI) class or CURB criteria. Data were extracted and classified by one author (MS) and entered into a Microsoft Access 2007 database.

### Statistical Analysis

Studies in which only two diagnostic tests were used were weighted the same as those studies in which three diagnostic tests were used, but these studies did not contribute to all proportions calculated. In cases in which zero positive cases were identified, we added 0.5 to the numerator and 1.0 to the denominator, as study weights (the inverse of the variance) would otherwise be undefined.

Studies were stratified by the proportion of study participants who received antecedent antibiotics into high and low categories. This proportion was not known for three studies. In two studies from Spain, the proportion was assumed to be the mean of the proportion of other studies from Spain. In a study among Navajo, the proportion was assumed to be low, and this study was categorized with other studies in which the proportion on antibiotics was low.

Severity of illness was defined using the Pneumonia Severity Index (PSI), which uses an algorithm to identify patients with CAP at low risk of dying within 30 days of presentation: a score of 1 represents the lowest risk and a score of 5 represents the highest risk [13]. In some cases, studies that did not record the patients' PSI class were characterized as having severe or non-severe populations based on the proportion in the ICU or the proportion hospitalized.

The methods and estimates were developed through an interactive process that included discussions with an independent Expert Review Panel as well as independent consultations with additional experts on CAP and pneumococcal pneumonia. Revisions were based on their comments and suggestions.

We sought to determine the proportion of pneumococcal pneumonia that is bacteremic, the proportion of CAP attributable to pneumococcus, and the contribution of the Binax UAT in diagnosing pneumococcal pneumonia over and beyond conventional culture techniques. Random effects meta-analysis to summarize the results was used because significant heterogeneity across studies was observed [14]. Analysis was done on the log-transformed study estimates. The numbers of study participants who underwent blood cultures, sputum cultures, or the Binax UAT were known; however, the numbers of people who underwent more than one diagnostic test were not (we knew that x people underwent blood culture and y people underwent sputum culture but not if the person who underwent blood culture was part of the group who underwent sputum culture). Thus, to calculate the proportion of CAP identified as pneumococcal, we used the total study population as the denominator, assuming all subjects were tested by all methods. We then evaluated the proportion of CAP identified as pneumococcus by blood culture, sputum culture, or the Binax UAT among those who actually underwent each test.

Sensitivity analysis was done by excluding outlying study values in order to determine their influence. We also stratified studies by proportion of pneumococcal pneumonia cases who were HIV-infected (<20% or unknown vs. ≥20%), severity of illness, and prior antibiotic use. Univariate linear regression (which assigns equal weights to studies) and random effects meta-regression were used to explore potential confounding factors for the proportion of CAP identified as pneumococcus. The Monte Carlo permutation test was applied to the meta-regression to obtain p-values adjusted for multiple testing [15]. A significance level of 0.05 was used to identify potential confounders between studies, which included hospital admission, HIV infection, antibiotics received before diagnostic testing, mean age, mean Pneumonia Severity Index (PSI), and whether concentrated urine was used for the Binax UAT testing. Finally, we analyzed the effect of (a) receipt of antibiotics before specimen collection and (b) PSI class comparing higher (IV–V) vs. lower (I–III) PSI classes on diagnostic yield. Heterogeneity of studies was evaluated by the I-squared test. Statistical analyses were done in Stata (version 11) [16].

## Results

**Figure 3:**
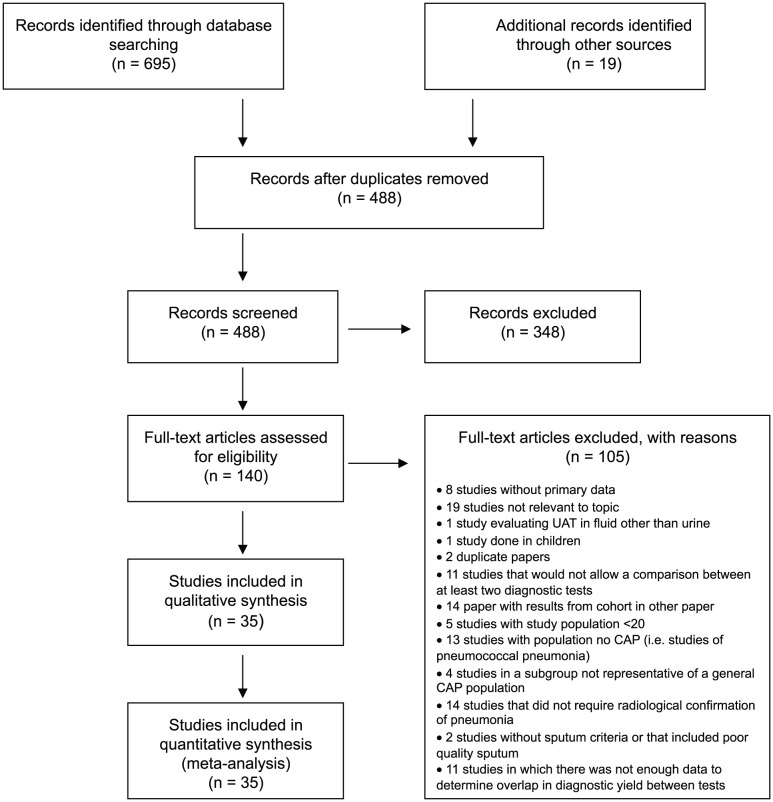
Flow diagram for the selection of studies.

Review of citations' titles and abstracts identified from a systematic literature search and hand searching of citation lists from other relevant published studies yielded 488 articles (Figure 3). Thirty-five studies reported the yield of the Binax UAT relative to blood culture and/or sputum culture in diagnosing adult pneumococcal pneumonia and were included in the analysis; of these, 28 had data for the relationship of positive results for all three diagnostic tests and were used in the calculations of the proportion of pneumococcal pneumonia that is bacteremic and the proportion of CAP attributable to pneumococcus. The included studies represented data from 18 countries (Table 1), predominantly from Europe and North America. The majority of included studies were prospective studies of CAP etiology among hospitalized cases.

**Table 1:** Studies included in the meta-analysis and their descriptors.

Study ID 1 2 3	Study First Author	Additional Data Obtained from Study Author	Country	CAP Study Size	Positive Results Among Those Undergoing Diagnostic Testing			Study Descriptors		
					Number of blood cultures positive for pneumococcus (% positive among tests performed)	Number of sputum cultures positive for pneumococcus (% positive among tests performed on good quality specimen)	Number of Binax UAT positive for pneumococcus (% positive among tests performed)	Proportion study participants who received antecedent antibiotics	Proportion study participants in PSI IV–V	Proportion study participants who were class known to be HIV infected
Europe										
1.	Mareković [39]	X	Croatia	80	0/11 (0)	8/36 (22)	16/76 (21)	30	40	0
2.	Krčová [40]	X	Czech Republic	84	2/84 (2)	3/45 (7)	11/84 (13)	0	Not known	0
3.	Lim [41]		England	267	9/225 (4)	9/73 (12)	69/214 (32)	39	Not known	0
4.	Hohenthal [42]	X	Finland	384	52/379 (14)	6/68 (9)	81/333 (24)	29	23	0
5.	Lasocki [43]		France	108	6/108 (6^4^)	18/36 (50)	34/108 (31)	70	0	0
6.	Endeman [44]	X	Netherlands	201	17/182 (9)	33/148 (22)	30/183 (16)	24	42	0
7.	Snijders [45]	X	Netherlands	213	16/208 (8)	18/67 (27)	32/211 (15)	24	44	0
8.	Van der Eerden [46]	X	Netherlands	262	30/254 (12)	15/44 (34)	52/262(20/)	26	44	0
9.	Hernes [47]	X	Norway	20	1/20 (5)	0/11 (0)	4/20 (20)	100	10	0
10.	Guchev [48]	X	Russia	219	N/A	25/73 (34)	48/219 (22)	11	Not known	0
11.	Beović [49]	X	Slovenia	109	1/109 (1)	3/22 (14)	13/109 (12)	30	0	0
12.	Andreo [50]	X	Spain	107	5/84 (6)	11/49 (22)	15/92 (16)	26	Not known	0
13.	Falguera [51]	X	Spain	3,413	383/3413 (11)	N/A	625/2252 (28)	25	45	0
14.	Falguera [52]	X	Spain	177	16/163 (10)	8/43 (19)	41/177 (23)	22	58	0
15.	Fernández-Sabé [53]	X	Spain	1,474	141/1416 (10)	205/583 (35)	27/88 (31)	25	56	0
16.	Gutiérrez [30]	X	Spain	493	13/302 (4)	14/272 (5)	104/454 (23)	23	25	<1
17.	Marcos [54]		Spain	398	45/398 (11)	64/183 (35)	109/398 (27)	Not known	Not known	21
18.	Ortega [32]	X	Spain	128	13/123 (11)	13/82 (16)	29/122 (24)	39	32	0
19.	Perelló [55]	X	Spain	64	10/64 (16)	17/64 (27)	31/64 (48)	0	Not known	100
20.	Sordé [56]	X	Spain	474	53/382 (14)	48/123 (39)	136/383 (36)	18	58	4
21.	Vidal [57]		Spain	555	37/555 (7)	80/284 (28)	140/555 (25)	Not known	12	0^5^
22.	Johansson [58]	X	Sweden	184	27/179 (15)	19/128 (15)	33/169 (20)	22	41	1
23.	Strålin [31]	X	Sweden	235	25/235 (11)	36/112 (32)	52/215 (24)	17	40	0
Asia										
24.	Ehara [59]	X	Japan	32	N/A	14/32 (44)	13/32 (41)	28	Not known	0
25.	Ishida [60]		Japan	349	12/349 (3)	72/349 (21^4^)	115/349 (33)	55	27	Not known
26.	Kobashi [61]		Japan	156	3/156 (2)	23/114 (20)	44/156 (28)	45	26	Not known
27.	Lauderdale [62]	X	Taiwan	168	5/168 (3)	13/168 (8)	35/168 (21)	16	Not known	Not known
Oceania										
28.	Charles [63]	X	Australia	885	33/868 (4)	40/524 (8)	95/850 (11)	30	54	-<1
29.	Weatherall [64]	X	Australia	59	0/41 (0)	3/19 (16)	9/59 (15)	25	40	0
30.	Murdoch [21]	X	New Zealand	474	22/443 (5)	63/332 (19)	120/420 (29)	27	50	0
Latin America										
31.	Díaz [65]	X	Chile	176	17/165 (10)	7/63 (11)	40/152 (26)	30	63	0
32.	Matute [66]	X	Nicaragua	130	N/A	4/49 (8)	5/67 (7)	0	Not known	0
North America										
33.	Butler [67]	X	U.S.A.	149	14/135 (10)	42/148 (28)	67/147^6^ (46)	62	Not known	0
34.	Nuermberger [68]	X	U.S.A.	487	40/408 (10)	47/168 (28)	98/399 (25)	20	24	44
35.	Watt et al. [69]	X	U.S.A.	68	3/41 (7)	4/27 (15)	9/53 (17)	Not known	Not known	Not known

1 Only patients with radiographically confirmed pneumonia were included.

2 All studies were prospective, except #5 and #13.

3 All studies were included in the analyses of the proportion of pneumococcal pneumonia that is bacteremic and the proportion of CAP attributable to pneumococcus except # 10, #24, and #32, because these did not include blood cultures, #13, because this did not include sputum cultures, and #21 and #25, because even though all three diagnostic tests were done, we did not have sufficient information to determine the overlap in diagnostic yield of the three tests.

4 The numbers of patients undergoing the test was unknown; thus, the percentage is the number of positive tests among all study participants.

5 Patients with HIV were excluded if CD4<200.

6 All specimens were collected within 12 hours of the first dose of antibiotics.

**Figure 4:**
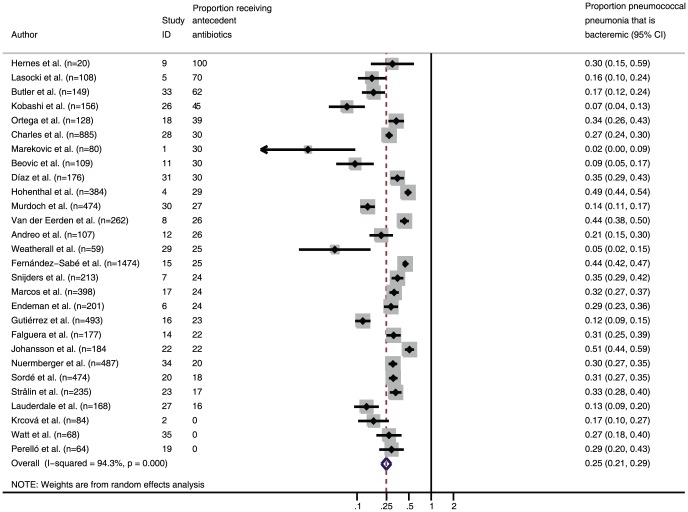
Forest plot for the proportion of pneumococcal pneumonia identified as bacteremic, sorted by proportion of study participants who received antecedent antibiotics.

**Figure 5:**
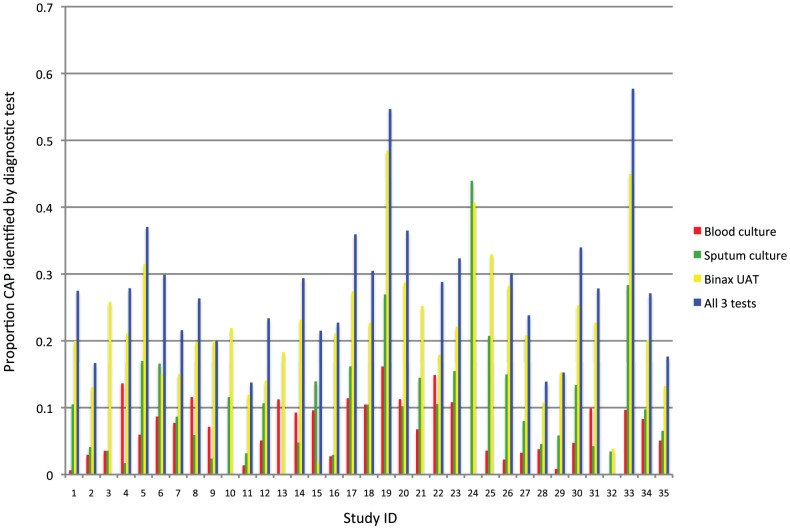
The proportion of community-acquired pneumonia attributable to pneumococcus, according to diagnostic test results, by individual studies^3^.

The proportion of pneumococcal pneumonia that was bacteremic ranged from 2.2% to 50.9% (median 28.9%, IQR 14.8% to 33.4%). In the meta-analysis, the proportion of pneumococcal pneumonia estimated to be bacteremic was 24.8% (95% CI: 21.3%, 28.9%) (Figure 4). The proportion of CAP identified as pneumococcus varied by diagnostic test. It was smallest with blood cultures and greatest with the Binax UAT (Figure 5). The meta-estimate of the proportion of CAP attributable to pneumococcus was 27.3% (95% CI: 23.9%, 31.1%). Inclusion criteria were used to maximize the quality of the studies included in the analysis. However, studies with larger population sizes were thought to provide more reliable proportions and were analyzed separately in order to assess this particular bias. When studies with <200 participants were compared with studies with ≥200 participants, the proportion of CAP attributable to pneumococcus was 27.4% (95% CI: 22.2%, 33.9%) in the smaller studies compared to 26.7% (95% CI: 22.7%, 31.4%) in the larger studies.

When we used as the denominator the number of people who actually underwent a diagnostic test rather than the total study sample size to evaluate the pneumococcal yield of each individual test, the proportion of CAP diagnosed as pneumococcal rose from 7.5% (95% CI: 6.4%, 8.8%) to 8.1% (95% CI: 7.0%, 9.5%) by blood culture, from 9.8% (95% CI: 8.1%, 12.0%) to 19.9% (95% CI: 16.9%, 23.4%) by sputum culture, and from 20.0% (95% CI: 17.4%, 23.1%) to 23.9% (95% CI: 21.7%, 26.4%) by the Binax UAT.

The Binax UAT increased the diagnostic yield over and above blood and sputum culture by an additional 11.4% (95% CI: 9.6%, 13.6%). Expressed as a ratio, the Binax UAT identified an additional 0.87 cases (95% CI: 0.51, 1.36) for every case identified as pneumococcal by blood and sputum culture.

We also stratified the results by prior antibiotic use, disease severity, and HIV prevalence (Table 2). In meta-regression, none of the included potential confounders were found to be significantly associated with proportion estimates. There was a high degree of heterogeneity in all outcomes of interest (e.g. I-squared for the proportion of CAP identified as pneumococcal by all three diagnostic tests was 91.6%), and none of the potential confounders included in the meta-regression explained this heterogeneity (adjusted p-values >0.05 for all covariates).

**Table 2:** Meta-analysis of the proportion of pneumococcal pneumonia that is bacteremic and the proportion of CAP attributable to pneumococcus, stratified by severity of disease, prior antibiotic use, and HIV status.

	Prior antibiotic use		Disease severity		HIV Prevalence	
Outcome Measure	High (n = 14)	Low (n = 14)	High (n = 12)	Low (n = 7)	High (n = 3)	Low (n = 21)
Proportion (%) of pneumococcal pneumonia that is bacteremic (95% CI)	20.1 (15.2–26.6)	28.4 (23.6–34.1)	31.3 (26.1–37.5)	20.6 (13.1–32.5)	30.9 (28.1–33.9)	26.2 (22.0–31.2)
Proportion (%) of CAP attributable to pneumococcus (95%CI)	26.4 (20.8–33.5)	28.0 (24.1–32.5)	27.3 (23.0–32.6)	25.8 (22.6–29.6)	37.3 (26.4–52.6)	25.9 (22.1–30.4)

We further investigated the effects of prior antibiotic use and PSI class (IV–V vs. I–III) on the diagnostic yield of each test and calculated the risk ratio for having a positive blood culture, sputum culture, or Binax UAT (Table 3). Prior antibiotics reduced the relative diagnostic yield for blood cultures by 67% (95% CI: 53%, 77%), for sputum cultures by 34% (95% CI: 8%, 53%) and for the Binax UAT by 26% (95% CI: 0%, 44%). The relative pneumococcal diagnostic yield among those people with more severe disease (PSI class IV–V) compared to those with less severe disease (PSI class I–III) increased for blood cultures by 72% (95% CI: 38%, 115%), and for the Binax UAT by 31% (95% CI: 15%, 50%).

**Table 3:** Meta-analysis of the association of prior antibiotic use and PSI class on the yield of blood culture, sputum culture, and the Binax UAT.

	Prior antibiotic use			Pneumonia Severity Index Class		
Diagnostic Test	Number of studies	Risk ratio^1^ (95% CI)	P-value	Number of studies	Risk ratio^2^ (95% CI)	P-value
Blood culture	17	0.33 (0.23–0.47)	<0.001	17	1.72 (1.38–2.15)	<0.001
Sputum culture	17	0.66 (0.47–0.92)	0.015	14	1.24 (0.98–1.56)	0.07
Binax UAT	17	0.74 (0.56–1.00)	0.047	16	1.31 (1.15–1.50)	<0.001

1 Of positive yield; reference is no prior antibiotic use.

2 Of positive yield; reference is Pneumonia Severity Index class I–III.

## Discussion

Through evaluation of the relationship in diagnostic yield of blood culture, sputum culture, and the Binax UAT in diagnosing adult pneumococcal pneumonia, we estimated the proportion of pneumococcal pneumonia that is bacteremic to be approximately 25% and, thus, the ratio of non-bacteremic pneumococcal pneumonia to bacteremic pneumococcal pneumonia to be approximately 3∶1. Because this estimate assumed a normal distribution, we also performed a bootstrap analysis [17] to determine the ratio of non-bacteremic pneumococcal pneumonia to bacteremic pneumococcal pneumonia, which gave a similar result. The wide range among studies in the proportion of pneumococcal pneumonia that was bacteremic could not be explained by study size, study location, proportion of participants undergoing sputum cultures and Binax UAT testing, proportion of participants on antecedent antibiotics, and severity of illness; however, in the two studies with the lowest proportion of bacteremic cases, many people did not undergo blood cultures and thus, some bacteremic cases were probably not identified. In the study with the most extreme value for this proportion (2.2%), only 11 of 80 study participants underwent blood culture testing, likely leading to a proportion underestimate.

This finding of a ratio of non-bacteremic to bacteremic pneumococcal pneumonia of 3∶1 is consistent with estimates from the pre-antibiotic era, which suggested that for every case of bacteremic pneumococcal pneumonia, there were 2–4 cases of non-bacteremic pneumococcal pneumonia [18], [19], [20]. Diagnosis at that time relied on extensive microbiological techniques and was unaffected by antibiotic use. Although our estimates are similar to those from the pre-antibiotic era, we used results from only three diagnostic tests, and the test with the highest yield, the Binax UAT, is known in cases of non-bacteremic pneumococcal pneumonia to have a sensitivity of as low as 52% among patients with sputum cultures positive for pneumococcus [21]. Furthermore, many patients in these etiologic studies received antecedent antibiotics, and not all patients underwent every diagnostic test. Thus, we believe that our estimate of the proportion of pneumococcal pneumonia that is bacteremic represents a lower limit of the true proportion.

We found that at least one-quarter of CAP cases are likely attributable to pneumococcus. This is consistent with a systematic review of 127 study cohorts among adults from 1966–1995 that found that pneumococcus accounted for 24% of all pneumonia cases [22]. Another source of validation is a study analyzing the impact of infant pneumococcal conjugate vaccination on U.S. pneumonia and influenza hospitalization and mortality rates for all ages. The study estimated, using modeling techniques, a reduction in the proportion of CAP attributable to pneumococcus from about 35–40% in the pre-vaccine era to about 18–28% in the post-vaccine era [23]. As the Binax UAT test was introduced around the same time as the pediatric pneumococcal conjugate vaccine, it is possible that the results from our analysis may reflect some indirect effects of the vaccine. Our estimate of the proportion of CAP attributable to pneumococcus is somewhat lower than the estimate for children; using a vaccine-probe approach, a meta-analysis of 4 randomized pediatric pneumococcal conjugate vaccine efficacy trials estimated the proportion of radiologically confirmed pneumonia attributable to pneumococcus to be 36% [2]. However, some of the pediatric vaccine trials were conducted in resource-limited countries and may explain the higher pneumococcal pneumonia burden compared to our findings among adults in high-resource settings.

All of the studies used to estimate the proportion of pneumococcal pneumonia that is bacteremic and the proportion of CAP attributable to pneumococcus by all 3 diagnostic methods were performed in resource-rich countries; the study from Nicaragua did not include data on the yield of blood cultures and, thus, did not contribute to these estimates. Given true differences in pneumonia epidemiology in resource-limited compared to resource-rich populations, such as age distribution, prevalence of nasopharyngeal pneumococcal colonization, the prevalence of underlying illness (e.g. HIV), and the ability to identify the etiology of CAP due to disparities in laboratory testing, access to care, and antecedent antibiotic use, it is possible that both the true and estimated proportion of pneumococcal pneumonia that is bacteremic and the proportion of CAP attributable to pneumococcus might differ. The paucity of data from resource-limited settings, particularly Africa, makes these differences difficult to assess. One recent study from Kenya, which used a serotype-specific latex agglutination urine antigen test, estimated by latent class analysis the proportion of CAP attributable to pneumococcus in adults to be 46% (95% CI: 36%–57%), over 18% higher than what we found [24].

Etiology studies among adults in resource-limited settings often use blood culture and sputum examination. By estimating the additional contribution of the Binax UAT in diagnosing pneumococcal pneumonia, we demonstrate that the Binax UAT may identify an additional 11% with an etiology of pneumococcal pneumonia compared to studies that only use blood culture and sputum examination. A recent study in South Africa of a rapid molecular assay for nasopharyngeal pneumococcal density in a population with high HIV prevalence found, similar to this study, that blood culture, good quality sputum culture and gram stain identified 15.4% of the population with pneumococcus and that the Binax UAT added an additional 11.7% [25].

The majority of studies included only hospitalized patients; only 5 studies included non-hospitalized patients. When we excluded these studies from the analysis, the results did not change meaningfully. The proportion of pneumococcal pneumonia that was bacteremic demonstrated an increasing trend in studies with more severe disease, which is consistent with findings that bacteremia is more common among patients with severe disease [26]. The proportion of pneumococcal pneumonia that was bacteremic was lower, although not statistically significantly lower, in studies in which a high proportion of the study population received antecedent antibiotics, likely indicating the greater effect antibiotic use has on blood culture yield compared to other diagnostic tests. The majority of studies included no patients with known HIV infection. Among three studies with an HIV prevalence ≥20%, a greater proportion of CAP was attributable to pneumococcus than among the other studies. This finding is consistent with studies that reported greater risk of developing IPD or bacterial pneumonia among HIV-infected individuals compared to the general population [27], [28].

We were able to further investigate, through a meta-analysis of risk ratios, the effect of antibiotic use and severity of illness on pneumococcal diagnostic test positivity. As expected, we found a decreased yield of blood culture and sputum culture among those who had received previous antibiotics. We also found a decreased yield of the Binax UAT after antibiotics, although the result was not statistically significant. Previous studies have shown conflicting results as to whether antecedent antibiotic use decreases Binax UAT yield [29], [30], [31]. We found increased yield of the three diagnostic tests with increased severity of disease, although for sputum culture this was not statistically significant. Many studies have shown that conventional diagnostic tests of blood culture and sputum culture have higher yield among sicker patients [26], [32], [33]. Studies among adults have demonstrated the Binax UAT to have a sensitivity of 77%–92% [21], [29], [34], [35], [36] in diagnosing bacteremic pneumococcal pneumonia and 52%–78% in diagnosing non-bacteremic pneumococcal pneumonia [21], [29], [34], [35], suggesting that the UAT is more sensitive in bacteremic patients; however the studies are difficult to compare as each defines non-bacteremic pneumococcal pneumonia differently. In some, but not all studies, the Binax UAT yield increased with higher PSI score [29], [30], [32].

There are a number of limitations to our study. Most notably, our findings are best generalizable to CAP patients presenting to hospital in developed countries. We only included studies in which the Binax UAT was used and thus were not able to include many studies from resource-limited countries. The direction of this bias depends on multiple factors and is difficult to predict. Higher HIV prevalence in some settings might lead to a higher true proportion of CAP attributable to pneumococcus, but a lack of adequate lab facilities in resource-limited settings might reduce the diagnostic yield and thus suggest a lower reported proportion of CAP attributable to pneumococcus. The studies included may also not be representative of the general CAP population, as almost all the patients were hospitalized and thus had moderate to severe disease and/or likely increased prevalence of comorbidities. An outpatient population with less severe disease would be expected to have a smaller proportion of pneumococcal pneumonia that was bacteremic than a hospitalized patient population, and thus, our estimate might underestimate the true proportion. By estimating the additional contribution of the Binax UAT over the conventional diagnostic tests (blood and sputum culture) and by estimating the degree to which antecedent antibiotic use and severity of illness is associated with diagnostic test yield, we aimed to provide a range of plausible estimates. For example, although the estimates here are representative of a hospitalized population in a resource-rich setting, the finding that the Binax UAT identified an additional 0.87 cases (95% CI: 0.51, 1.36) for every case identified as pneumococcal by blood or sputum culture could be used when estimating the burden of pneumococcal pneumonia in populations in which the Binax UAT was not used.

We were also limited by the fact that not all patients underwent all diagnostic tests. Blood cultures were performed on 14%–100% (median 97% and mean 89%) of participants. Sputum cultures were performed on 20%–100% of participants (median 60% and mean 60%); and the Binax UAT was performed on 6%–100% (median 96% and mean 90%) of participants. By assuming that all participants underwent each test, we underestimated the proportion of people infected with pneumococcus. Because sputum cultures were performed on fewer people than blood cultures, when we calculated the proportion of CAP cases diagnosed as pneumococcal by each diagnostic test using the true number of study participants who underwent each test, rather than all study participants, the yield of sputum culture rose significantly; thus, this limitation may have led to an overestimation of the proportion of pneumococcal pneumonia that is bacteremic. Furthermore, we do not know whether those people who underwent certain diagnostic tests were qualitatively different than those who did not undergo certain diagnostic tests. If blood cultures were collected more often by some clinicians than others, but urine was collected on all subjects, this might bias our results to underestimate the proportion of pneumococcal pneumonia that is bacteremic.

Finally, our results are constrained by the limitations in sensitivity and specificity of the diagnostic tests themselves. For example, assuming that the Binax UAT is more sensitive among bacteremic patients, it might miss a larger number of non-bacteremic patients and lead to a falsely high proportion of pneumococcal pneumonia thought to be bacteremic.

Our study shows that estimates of the number of pneumococcal pneumonia adult cases that rely primarily on blood culture results vastly underestimate the true burden of disease. The objective of this analysis was not to conduct a meta-analysis of the tests' performances but to provide a range of plausible values in order to estimate, with tests known to be imperfect, the burden of non-bacteremic pneumococcal pneumonia among adults. Additional studies utilizing the Binax UAT and development and use of more reliable tests for non-bacteremic pneumococcal pneumonia, including possibly quantitative sputum or nasopharyngeal PCR, will improve our understanding. The observed reduction in both IPD and pneumococcal pneumonia among unvaccinated older children and adults due to the indirect effects of pediatric conjugate vaccine can also provide a measure of pneumococcal pneumonia burden [23], [37], [38]. Through this and additional studies, a better understanding of the adult disease burden can help guide treatment and prevention strategies.

## Supporting Information

Figure S1 **The relationship between diagnostic testing and the proportion of CAP attributable to pneumococcus.**
(EPS)

Appendix S1 **Estimating the burden of pneumococcal pneumonia among adults: the conceptual framework.**
(DOCX)
