# Supplementary material for: Estimating the Burden of Pneumococcal Pneumonia among Adults: A Systematic Review and Meta-Analysis of Diagnostic Techniques
Source: PLoS One. 2013 Apr 2;8(4):e60273. doi: 10.1371/journal.pone.0060273 (PMC3615022; doi:10.1371/journal.pone.0060273)
Supplement: Appendix S1 — Estimating the burden of pneumococcal pneumonia among adults: the conceptual framework. (DOCX) [file pone.0060273.s002.docx]

**Title:**  Estimating the burden of pneumococcal pneumonia among adults: the conceptual framework

**Running Title**: Adult pneumococcal pneumonia burden

**Authors:** Maria A. Said^1^, MD, MHS, Hope L. Johnson², PhD, MPH, Maria Deloria-Knoll², PhD, Katherine L. O’Brien^²^, MD, MPH

**Affiliations**

1. Department of Medicine, Division of Infectious Diseases, Johns Hopkins School of Medicine, Baltimore, MD, USA
2. International Vaccine Access Center, Johns Hopkins Bloomberg School of Public Health, Baltimore, MD, USA

**Corresponding Author:**

Maria Said, MD, MHS

201 W. Preston Street

Baltimore, MD 21201

Phone: 410-767-7395

Fax: 410-669-4215

said.maria@gmail.com

**Grant Support:** The Bill and Melinda Gates Foundation, NIH T32 Training Grant

**Conflict of Interest Statements:** None

**Keywords:** pneumococcus, *Streptococcus pneumoniae*, pneumonia, Binax, disease burden

**Abstract Word Count:** 254

**Text Word Count:** 2,927

**References:** 40

**Tables:** 0

**Figures:** 1

**Abstract**

***Background***. The adult global burden of pneumococcal pneumonia is not well quantified, and lack of a gold standard test for non-bacteremic pneumococcal pneumonia limits estimation attempts. Our objective was to develop a conceptual framework and modeling strategy to estimate the burden of non-bacteremic pneumococcal pneumonia as part of a larger project aimed at estimating the pneumococcal disease burden among adults.

***Methods and Findings.*** We reviewed the literature to better understand the limitations of available diagnostic tests, how pneumococcal pneumonia is diagnosed, and how multiple studies’ results could be compared. The model utilizes the relative yield of the Binax *S. pneumoniae* urinary antigen test (UAT), blood cultures, and sputum cultures to estimate the proportion of pneumococcal pneumonia that is bacteremic (and thus the ratio of non-bacteremic to bacteremic pneumococcal pneumonia) and the proportion of community-acquired pneumonia (CAP) attributable to pneumococcus. These values can then be anchored on the incidence of bacteremic pneumococcal pneumonia and the incidence of all-cause CAP as reported in the literature in order to estimate the burden of non-bacteremic pneumococcal pneumonia among adults. This model serves as the basis for a systematic literature review and meta-analysis of diagnostic techniques. We also discuss the process of model development and predict the likely direction and magnitude of biases inherent in the outcome estimates.

***Conclusions***. Although little literature exists on the burden of non-bacteremic pneumococcal pneumonia among adults, it is possible, through the use of this model, to estimate its burden, thus providing a better understanding of the overall global burden of pneumococcal disease among adults in order to guide future research and policy decisions.

**Introduction**

Community-acquired pneumonia (CAP) is a significant cause of morbidity and mortality worldwide, of which a large proportion is caused by *Streptococcus pneumoniae* (pneumococcus). Estimates of the burden of pneumococcal pneumonia among children found almost 14 million cases in the year 2000 and 485,000 deaths in the year 2008 {O'Brien, 2009 #7;World Health Organization, 2012 #4179}. Among those ≥15 years of age, lower respiratory tract infections are thought to be the third leading cause of death {World Health Organization (WHO), 2008 #4077}, but the global burden of pneumococcal pneumonia among adults is not known.

The ability to quantify the burden of pneumococcal pneumonia is limited not only by the likelihood that people with disease will seek medical care and the degree of testing applied to them, but also by the characteristics of the diagnostic tests. There is no gold standard to diagnose non-bacteremic pneumococcal pneumonia, and available diagnostic tests have limitations. Given the high specificity of blood culture and other invasive tests compared to sputum culture, most pneumonia etiology studies traditionally have measured only the incidence of invasive pneumococcal disease (IPD) or bacteremic pneumococcal pneumonia. Therefore, these studies underestimate the true burden of disease. As part of a larger project aimed at estimating the global burden of pneumococcal disease among adults, we developed a model by which we could estimate the burden of non-bacteremic pneumococcal pneumonia. The process of model development and the limitations of the outcome estimates are described here.

**Initial Model Development**

An exploratory literature review was conducted of over 100 studies containing information on the burden of pneumococcal pneumonia, with no limits placed on study location or study time period, to better understand the data available. Studies included CAP etiology and incidence studies, evaluation of diagnostic tests, development of clinical prediction rules, drug efficacy trials, vaccine effectiveness studies, and management and outcome studies. Particular attention was placed on identifying the range of diagnostic tests used, the limitations of these tests, the ways pneumococcal pneumonia was defined, and whether and how the results from disparate studies could be compared.

Because there is no gold standard, studies used a range of different diagnostic tests to diagnose pneumococcal pneumonia, some of which, such as capsular antigen detection by countercurrent immunoelectrophoresis, are no longer routinely used. Blood cultures, while highly specific, are positive in only a small proportion of cases {Waterer, 2001 #4038}. The yield of sputum gram stain and culture is limited by the inability of many patients to produce adequate quality sputum samples {Ewig, 2002 #3918;Lentino, 1987 #3872;Garcia-Vazquez, 2004 #3662;Musher, 2004 #3875}, and specificity of culture is compromised by contamination of the upper airways by colonizing bacterial flora {Bartlett, 1998 #2379}. Invasive diagnostic techniques such as transtracheal aspiration, transthoracic aspiration, and bronchoscopy can increase the yield beyond that from blood culture and are very specific, but the sensitivity of these tests depends on pneumococcal organisms existing within fluid that is abstracted and they are not applicable in many clinical settings. Furthermore, even in settings that conduct these procedures, only a small proportion of patients with CAP will undergo these invasive diagnostic tests and those who do are likely to be different than those who do not. Analysis of antibody titers in paired sera, although useful for diagnosing infection for some pathogens, has not been useful for pneumococcal infection because no test with acceptable sensitivity and specificity has been developed, given the diversity of pneumococcal capsular polysaccharides {Kalin, 1985 #4142}.

Antigen detection methods, including by countercurrent-immunoelectrophoresis and latex agglutination, of urine or respiratory specimens have been used, especially in European studies, but are thought to be limited by cost, time, or lack of sensitivity and specificity {Bartlett, 1998 #2379} and are therefore not part of current guidelines for pneumonia diagnosis. The BinaxNOW^®^ *S. pneumoniae* urinary antigen test (UAT) (Binax, Inc. Inverness Medical, Scarborough, Maine, USA), a non-serotype-specific test, was licensed by the U.S. FDA in 1999 and offers an additional means of diagnosing both bacteremic and non-bacteremic pneumococcal pneumonia. Studies among adults have demonstrated the Binax UAT to have a sensitivity of 77%-92% {Domínguez, 2001 #957;Murdoch, 2001 #1638;Gutierrez, 2003 #2746;Smith, 2003 #1598;Roson, 2004 #2064} and 52%-78% {Domínguez, 2001 #957;Murdoch, 2001 #1638;Gutierrez, 2003 #2746;Roson, 2004 #2064} in diagnosing bacteremic and non-bacteremic pneumococcal pneumonia, respectively, with a specificity of >90% compared to patients with bacteremia or pneumonia caused by another organism {Domínguez, 2001 #957;Smith, 2003 #1598}. Concentrating the urine increased assay sensitivity in some studies {Marcos, 2003 #2742} but is not done routinely {Murdoch, 2001 #1638}. The assay can also produce positive results in people recently recovered from pneumococcal infection {Marcos, 2003 #2742}. While the Binax UAT is fast and simple to use, its cost (approximately $30 per specimen) may limit widespread use {Mandell, 2007 #2330}.

PCR remains a potential test of interest; however, pneumococcal PCR methods have not been standardized and at this time are not recommended for routine diagnostic use. A recent meta-analysis of 29 studies investigating the utility of pneumococcal PCR on blood demonstrated a sensitivity of 57.1% among patients with pneumococcal bacteremia and a specificity 98.6% comparing pneumococcal bacteremia patients with healthy people or those who had bacteremia caused by other organisms {Avni, 2010 #2559}. Sputum pneumococcal PCR, while demonstrating high sensitivity and good results seen even after antibiotic use {Johansson, 2008 #3999}, is limited by low specificity for pneumococcal pneumonia diagnosis due to detection of colonization rather than infection {Murdoch, 2003 #2014} and identification of gene targets found also in other bacteria that can colonize the mouth {Whatmore, 2000 #3997}.

Not only did studies use different diagnostic tests by which to diagnose pneumococcal pneumonia, but they also defined pneumococcal pneumonia variably. Some used criteria to categorize cases as “definite” and “probable”; others did not. Given the differences in diagnostic tests used and the ways in which pneumococcal pneumonia was defined in each study, we could not directly compare the outcome measures describing the burden of bacteremic and non-bacteremic pneumococcal pneumonia from each study. A way to compare results from disparate studies was needed.

**Conceptual framework for estimating the burden of pneumococcal disease in adults**

We developed a conceptual framework to describe the relationships between bacteremic pneumococcal pneumonia, non-bacteremic pneumococcal pneumonia, and all-cause CAP. We then developed a model by which we could leverage what is known about bacteremic pneumococcal disease and CAP in order to better understand the burden of non-bacteremic pneumococcal pneumonia, for which there is less empiric data.

The model uses the incidence of bacteremic pneumococcal pneumonia, as reported in the literature, as an anchor and estimates the absolute burden of adult bacteremic pneumococcal pneumonia cases by applying total global adult population estimates to these incidence values. The non-bacteremic pneumonia disease burden can be determined by multiplying the absolute burden of bacteremic pneumonia by the ratio of non-bacteremic to bacteremic pneumococcal pneumonia cases. The sum of the bacteremic and non-bacteremic cases makes up the total pneumococcal pneumonia disease burden estimates for adults.

As a plausibility check, we developed a second conceptual model in which we anchored the burden of pneumococcal pneumonia (both bacteremic and non-bacteremic) on an estimate of the incidence of all-cause CAP. In this approach, we multiplied the estimated number of CAP cases (derived from incidence and total adult population values) within a community by the proportion of CAP attributable to pneumococcus.

These two strategies require that we know (a) the ratio of non-bacteremic pneumococcal pneumonia to bacteremic pneumococcal pneumonia and (b) the proportion of CAP attributable to pneumococcus.

**Model Flexibility**

Given the variations in sensitivity and specificity of the available pneumococcal diagnostic tests and the variability of their use in studies, the values determined for the ratio of non-bacteremic to bacteremic pneumococcal pneumonia or the proportion of CAP attributable to pneumococcus depends on the type and extent to which diagnostic tests are used. As increasing numbers of diagnostic tests are used, the estimated ratio of non-bacteremic to bacteremic pneumococcal pneumonia and the estimated proportion of CAP that can be attributed to pneumococcus become greater.

In Scenario 1 (Figure S1), when blood culture alone is used to diagnose pneumococcal pneumonia, the estimated proportion of CAP attributable to pneumococcus is only 10%. In Scenario 5, in which positive results from invasive testing, sputum culture, the Binax UAT, and sputum PCR are added to the estimate derived from blood culture, the estimated proportion of CAP attributable to pneumococcus is greater than 50%. The estimated amount of non-bacteremic pneumococcal pneumonia also varies depending on the number of diagnostic tests used, thus affecting the estimated ratio of non-bacteremic to bacteremic pneumococcal pneumonia. Given the fact that blood culture lacks sensitivity and sputum PCR lacks specificity, the true values of this ratio and this proportion likely lie between these estimates.

Most etiology studies use blood cultures and sputum culture in the diagnosis of pneumococcal pneumonia. The Binax UAT has become increasingly utilized since its licensure, especially in developed countries due to its high specificity, relatively high sensitivity, and ease of use. Sputum PCR and invasive diagnostic testing, on the other hand, are rarely performed, and result from invasive testing, which is performed only in select cases, are likely qualitatively different than those from general CAP cases. Assuming, based on recent literature, that the proportion of CAP attributable to pneumococcus is <50%, a high test specificity is more important than a high sensitivity in preventing disease misclassification {Campbell, 2008 #3600}, and therefore the Binax UAT both offers the best estimate of the true burden of disease compared to the other methods evaluated. For these reasons, blood culture, sputum cultures, and the Binax UAT, but not invasive tests or sputum PCR, were included in our model.

**The Model**

Thus, we ultimately chose to include in our model results from the literature on the burden of pneumococcal pneumonia as diagnosed by blood culture, sputum culture, and the Binax UAT. In order to populate our model with data on the yield of blood culture, sputum cultures, and the Binax UAT, we conducted a comprehensive literature review to identify all studies that utilized the Binax UAT in addition to blood culture and or sputum culture. The raw numbers of the yield of these three individual diagnostic tests were extracted in order to understand the relative diagnostic yield of each test. Bacteremic pneumococcal pneumonia was defined as cases of radiographic pneumonia in which a blood culture was positive for pneumococcus. Non-bacteremic pneumococcal pneumonia was defined as cases of radiographic pneumonia in which there was positive Binax UAT and/or a sputum culture positive for pneumococcus but without a blood culture positive for pneumococcus. The proportion of CAP attributable to pneumococcus was defined as the proportion of cases in which at least one of these three diagnostic tests was positive. By pooling the results of multiple studies in a meta-analysis, we were able to determine an estimate for the proportion of pneumococcal pneumonia that is bacteremic (and thus the ratio of non-bacteremic pneumococcal pneumonia to bacteremic pneumococcal pneumonia) and the proportion of CAP attributable to pneumococcus (Said, MA, et al. Estimating the burden of pneumococcal pneumonia among adults: a meta-analysis of diagnostic techniques.).

**Model Limitations**

This model allows us the ability to compare the results of multiple different studies to better understand the ratio of non-bacteremic to bacteremic pneumococcal pneumonia and the proportion of CAP attributable to pneumococcus. However, estimations of the desired outcome measures are limited both by true differences between different study populations and the ability of the individual studies to ascertain these differences.

***Factors that affect the true proportion of pneumococcal pneumonia that is bacteremic***

A number of study population characteristics, including age distribution, prevalence of underlying illness, antibiotic use, severity of illness, and pneumococcal serotype could affect the proportion of pneumococcal pneumonia that is bacteremic. Whether age affects the risk of someone with pneumococcal infection becoming bacteremic is not clear. Some comparative studies found no significant age differences between those with bacteremic pneumococcal pneumonia and those with non-bacteremic disease {Musher, 2000 #815}; others found that on average, bacteremic patients were younger than non-bacteremic patients, although this could be attributed to higher rates of certain underlying conditions, such as HIV infection or intravenous drug use {Jover, 2008 #862}. Some studies have shown that the prevalence of certain underlying conditions are different in bacteremic and non-bacteremic patients; for example, patients with bacteremic pneumococcal pneumonia were, more likely to abuse alcohol, smoke, use intravenous drugs, have underlying cardiovascular disease and be infected with HIV {Jover, 2008 #862;Musher, 2000 #815;Ruiz, 1999 #2072;Ortqvist, 1988 #3876}. Bacteremia is also more common with more severe pneumonia disease {Waterer, 2001 #4038}. A prospective study in Sweden showed that with increasing pneumonia severity score (CURB score), the frequency of bacteremic pneumococcal pneumonia rose in spite of a constant risk of pneumococcal pneumonia across scores, leading to the inference that severity of illness does not so much affect the frequency of pneumococcal infection but rather its likelihood of invasion {Stralin, 2010 #4021}. Finally, some pneumococcal serotypes cause invasive disease more often than others {Brueggemann, 2004 #3637}, and the distribution and frequency of serotypes varies geographically {Johnson, 2010 #4068}.

***Factors that affect the true proportion of CAP attributable to pneumococcus***

To our knowledge, there are no clear data to indicate that the proportion of CAP due to pneumococcus varies by age. Among CAP etiology studies, the distribution of pathogens varies, but many studies find pneumococcus to be the most common etiologic agent across all age groups {Stralin, 2010 #4021;Lim, 2001 #1642}. Certain underlying conditions have been shown to place persons at risk for pneumococcal infection, including dementia, seizure disorders, cigarette smoking, congestive heart failure, cerebrovascular disease, institutionalization, and chronic obstructive pulmonary disease {Lipsky, 1986 #252}. Thus, a population of CAP patients in which the number of risk factors for pneumococcal infection is high might be expected to have a different proportion of pneumococcal pneumonia than a population with fewer underlying risk factors. There are no clear data, to our knowledge, to suggest that the relative proportion of CAP caused by pneumococcus varies by severity of illness; a review of 41 prospective etiology studies in Europe found *S. pneumoniae* to be the most common etiological agent in the community, the hospital, and the ICU setting {Woodhead, 2002 #4036}. Seasonality might affect the proportion; studies have shown peak incidence of IPD to occur during the winter months {Dowell, 2003 #4043}, but all-cause CAP also increases in incidence during these times. The relative proportion of CAP attributable to pneumococcus might vary by geography, although very little comparative data exists. No known comprehensive studies of the etiology of CAP among adults worldwide have been done; a study of the etiology among children is currently underway {Levine, 2012 #4151}. We do know that pneumococcal carriage rates varies greatly by location {Cardozo, 2006 #4126}, and thus, it is conceivable that the relative proportion of CAP attributable to pneumococcus might vary as well.

***Factors that affect investigators’ ability to determine the burden of pneumococcal pneumonia***

The ability to detect the true ratio of non-bacteremic to bacteremic pneumococcal pneumonia and the proportion of CAP attributable to pneumococcus is limited by the characteristics of the diagnostic tests and the degree to which they are used. None of the utilized diagnostic tests is 100% sensitive or specific, and study population factors can affect the performances of the tests. The yield of culture and the Binax UAT has been shown in some studies to increase with severity of illness {Ortega, 2005 #4028}, and the Binax UAT is more sensitive among bacteremic than non-bacteremic patients {Domínguez, 2001 #957}. Lower yield in these tests with less severe disease, then, might not only represent less pneumococcal disease but also a failure of the test to detect true disease. Antibiotics have been shown to reduce the yield of culture {Ewig, 2002 #3918} and might affect, although to a lesser extent, the yield of the Binax UAT {Smith, 2003 #1598;Roson, 2004 #2064}.

The degree to which these imperfect tests are utilized also affects investigators’ abilities to detect true disease. Not all patients within a study undergo all diagnostic tests; a study of routine microbial investigation in England found that of 122 patients admitted, diagnostic testing was often not done, with 81% undergoing blood culture, 45% undergoing sputum culture examination, and 28% undergoing complete serological tests {Woodhead, 1991 #3958}. Diagnostic test conduction and interpretation differences can also affect the results. The sensitivity of blood cultures, for example, depends on the choice of culture media, the duration of incubation, and the volume of blood collected. A study in Malawi found that improvements in culturing techniques led to a disproportionate increase in the yield of pneumococcus compared to other organisms, demonstrating that the pneumococcus is especially susceptible to poor collection practices {Mtunthama, 2008 #3601}. In addition, the pneumococcus does not survive well in broth cultures in hot climates {Siberry, 2001 #3627}, making culture less reliable in places without regulated temperature control. While automated detection methods can help, many microbiology laboratories in resource-poor settings are not staffed around the clock, and there may be a delay between signal of growth and sub-culturing leading to autolysis and false negative blood cultures. Problems with diagnostic testing are not confined to resource-limited countries; Bartlett argues that the decreasing ability to identify causative pathogens in the United States lies partly in changes in microbiological practices as a result of the Clinical Laboratory Improvement Act regulations and the use of empiric broad-spectrum antibiotics. {Bartlett, 2004 #1574}.

**Conclusion**

A conceptual framework and model, which would utilize results from the published literature to determine (a) the ratio of non-bacteremic pneumococcal pneumonia to bacteremic pneumococcal pneumonia and (b) the proportion of CAP attributable to pneumococcus, was developed to estimate the burden of non-bacteremic pneumococcal pneumonia. Because results from the literature are difficult to compare given differences in diagnostic tests used and in the ways pneumococcal pneumonia is defined, we determined to extract the raw numbers on the yield of three individual diagnostic tests: blood culture, sputum culture, and the Binax urine antigen test in order to compare the results of disparate studies. Our estimates have limitations, but despite these limitations, this strategy can provide a plausible estimate of the burden of pneumococcal pneumonia. These multiple approaches can serve to better inform future estimates of the burden of adult pneumococcal pneumonia, most of which is non-bacteremic in nature, and thus assist in setting priorities in treatment and prevention efforts.

**Figures**

Figure S1. The relationship between diagnostic testing and the proportion of CAP attributable to pneumococcus

* Red shaded bars represent cases identified as bacteremic pneumococcal pneumonia by blood culture. Blue shaded bars represent cases identified as non-bacteremic pneumococcal pneumonia by additional diagnostic tests. Each addition of a blue stacked bar indicates the additional cases identified by that method over and above those identified by the more specific tests below it. UAT = Urinary antigen test. Invasive testing includes transthoracic aspiration, transtracheal aspiration, and bronchoscopy.

**References**

1. O'Brien KL, Wolfson LJ, Watt JP, Henkle E, Deloria-Knoll M, et al. (2009) Burden of disease caused by Streptococcus pneumoniae in children younger than 5 years: global estimates. Lancet 374: 893-902.

2. World Health Organization (2012) Estimated Hib and pneumococcal deaths for children under 5 years of age, 2008.

3. World Health Organization (WHO) (2008) The Global Burden of Disease: 2004 Update. Geneva: WHO.

4. Waterer GW, Wunderink RG (2001) The influence of the severity of community-acquired pneumonia on the usefulness of blood cultures. Respir Med 95: 78-82.

5. Ewig S, Schlochtermeier M, Goke N, Niederman MS (2002) Applying sputum as a diagnostic tool in pneumonia: limited yield, minimal impact on treatment decisions. Chest 121: 1486-1492.

6. Lentino JR, Lucks DA (1987) Nonvalue of sputum culture in the management of lower respiratory tract infections. J Clin Microbiol 25: 758-762.

7. Garcia-Vazquez E, Marcos MA, Mensa J, de Roux A, Puig J, et al. (2004) Assessment of the usefulness of sputum culture for diagnosis of community-acquired pneumonia using the PORT predictive scoring system. Arch Intern Med 164: 1807-1811.

8. Musher DM, Montoya R, Wanahita A (2004) Diagnostic value of microscopic examination of Gram-stained sputum and sputum cultures in patients with bacteremic pneumococcal pneumonia. Clin Infect Dis 39: 165-169.

9. Bartlett JG, Breiman RF, Mandell LA, File Jr TM (1998) Community-acquired pneumonia in adults: Guidelines for management. Clinical Infectious Diseases 26: 811-838.

10. Kalin M, Lindberg AA (1985) Antibody response against the type specific capsular polysaccharide in pneumococcal pneumonia measured by enzyme linked immunosorbent assay. Scandinavian Journal of Infectious Diseases 17: 25-32.

11. Domínguez J, Galí N, Blanco S, Pedroso P, Prat C, et al. (2001) Detection of Streptococcus pneumoniae antigen by a rapid immunochromatographic assay in urine samples. Chest 119: 243-249.

12. Murdoch DR, Laing RT, Mills GD, Karalus NC, Town GI, et al. (2001) Evaluation of a rapid immunochromatographic test for detection of Streptococcus pneumoniae antigen in urine samples from adults with community-acquired pneumonia. J Clin Microbiol 39: 3495-3498.

13. Gutierrez F, Masía M, Rodriguez JC, Ayelo A, Soldan B, et al. (2003) Evaluation of the immunochromatographic binax NOW assay for detection of Streptococcus pneumoniae urinary antigen in a prospective study of community-acquired pneumonia in Spain. Clinical Infectious Diseases 36: 286-292.

14. Smith MD, Derrington P, Evans R, Creek M, Morris R, et al. (2003) Rapid diagnosis of bacteremic pneumococcal infections in adults by using the Binax NOW Streptococcus pneumoniae urinary antigen test: a prospective, controlled clinical evaluation. J Clin Microbiol 41: 2810-2813.

15. Roson B (2004) Contribution of a urinary antigen assay (Binax NOW) to the early diagnosis of pneumococcal pneumonia. Clin Infect Dis 38: 222-226.

16. Marcos MA, de Anta MTJ, de la Bellacasa JP, Gonzalez J, Martinez E, et al. (2003) Rapid urinary antigen test for diagnosis of pneumococcal community-acquired pneumonia in adults. European Respiratory Journal 21: 209-214.

17. Mandell LA, Wunderink RG, Anzueto A, Bartlett JG, Campbell GD, et al. (2007) Infectious Diseases Society of America/American Thoracic Society Consensus Guidelines on the management of community-acquired pneumonia in adults. Clinical Infectious Diseases 44: S27-S72.

18. Avni T, Mansur N, Leibovici L, Paul M (2010) PCR Using Blood for Diagnosis of Invasive Pneumococcal Disease: Systematic Review and Meta-Analysis. Journal of clinical microbiology 48: 489-496.

19. Johansson N, Kalin M, Giske CG, Hedlund J (2008) Quantitative detection of Streptococcus pneumoniae from sputum samples with real-time quantitative polymerase chain reaction for etiologic diagnosis of community-acquired pneumonia. Diagn Microbiol Infect Dis 60: 255-261.

20. Murdoch DR, Anderson TP, Beynon KA, Chua A, Fleming AM, et al. (2003) Evaluation of a PCR assay for detection of Streptococcus pneumoniae in respiratory and nonrespiratory samples from adults with community-acquired pneumonia. J Clin Microbiol 41: 63-66.

21. Whatmore AM, Efstratiou A, Pickerill AP, Broughton K, Woodard G, et al. (2000) Genetic relationships between clinical isolates of Streptococcus pneumoniae, Streptococcus oralis, and Streptococcus mitis: characterization of "Atypical" pneumococci and organisms allied to S. mitis harboring S. pneumoniae virulence factor-encoding genes. Infect Immun 68: 1374-1382.

22. Campbell H, Biloglav Z, Rudan I (2008) Reducing bias from test misclassification in burden of disease studies: use of test to actual positive ratio--new test parameter. Croat Med J 49: 402-414.

23. Musher DM, Alexandraki I, Graviss EA, Yanbeiy N, Eid A, et al. (2000) Bacteremic and nonbacteremic pneumococcal pneumonia. A prospective study. Medicine (Baltimore) 79: 210-221.

24. Jover F, Cuadrado JM, Andreu L, Martinez S, Canizares R, et al. (2008) A comparative study of bacteremic and non-bacteremic pneumococcal pneumonia. Eur J Intern Med 19: 15-21.

25. Ruiz M, Ewig S, Marcos MA, Martinez JA, Arancibia F, et al. (1999) Etiology of community-acquired pneumonia: impact of age, comorbidity, and severity. Am J Respir Crit Care Med 160: 397-405.

26. Ortqvist A, Grepe A, Julander I, Kalin M (1988) Bacteremic pneumococcal pneumonia in Sweden: clinical course and outcome and comparison with non-bacteremic pneumococcal and mycoplasmal pneumonias. Scand J Infect Dis 20: 163-171.

27. Stralin K, Olcen P, Tornqvist E, Holmberg H (2010) Definite, probable, and possible bacterial aetiologies of community-acquired pneumonia at different CRB-65 scores. Scand J Infect Dis 42: 426-434.

28. Brueggemann AB, Peto TE, Crook DW, Butler JC, Kristinsson KG, et al. (2004) Temporal and geographic stability of the serogroup-specific invasive disease potential of Streptococcus pneumoniae in children. J Infect Dis 190: 1203-1211.

29. Johnson HL, Deloria-Knoll M, Levine OS, Stoszek SK, Freimanis Hance L, et al. (2010) Systematic evaluation of serotypes causing invasive pneumococcal disease among children under five: the pneumococcal global serotype project. PLoS Med 7.

30. Lim WS, Macfarlane JT, Boswell TC, Harrison TG, Rose D, et al. (2001) Study of community acquired pneumonia aetiology (SCAPA) in adults admitted to hospital: implications for management guidelines. Thorax 56: 296-301.

31. Lipsky BA, Boyko EJ, Inui TS, Koepsell TD (1986) Risk factors for acquiring pneumococcal infections. Arch Intern Med 146: 2179-2185.

32. Woodhead M (2002) Community-acquired pneumonia in Europe: causative pathogens and resistance patterns. Eur Respir J Suppl 36: 20s-27s.

33. Dowell SF, Whitney CG, Wright C, Rose CE, Jr., Schuchat A (2003) Seasonal patterns of invasive pneumococcal disease. Emerg Infect Dis 9: 573-579.

34. Levine OS, O'Brien KL, Deloria-Knoll M, Murdoch DR, Feikin DR, et al. (2012) The pneumonia etiology research for child health project: a 21st century childhood pneumonia etiology study. Clinical infectious diseases : an official publication of the Infectious Diseases Society of America 54 Suppl 2: S93-S101.

35. Cardozo DM, Nascimento-Carvalho CM, Souza FR, Silva NM (2006) Nasopharyngeal colonization and penicillin resistance among pneumococcal strains: a worldwide 2004 update. Braz J Infect Dis 10: 293-304.

36. Ortega L, Sierra M, Dominguez J, Martinez J, Matas L, et al. (2005) Utility of a pneumonia severity index in the optimization of the diagnostic and therapeutic effort for community-acquired pneumonia. Scand J Infect Dis 37: 657-663.

37. Woodhead MA, Arrowsmith J, Chamberlain-Webber R, Wooding S, Williams I (1991) The value of routine microbial investigation in community-acquired pneumonia. Respir Med 85: 313-317.

38. Mtunthama N, Gordon SB, Kusimbwe T, Zijlstra EE, Molyneux ME, et al. (2008) Blood culture collection technique and pneumococcal surveillance in Malawi during the four year period 2003-2006: an observational study. BMC Infect Dis 8: 137.

39. Siberry G, Brahmadathan KN, Pandian R, Lalitha MK, Steinhoff MC, et al. (2001) Comparison of different culture media and storage temperatures for the long-term preservation of Streptococcus pneumoniae in the tropics. Bull World Health Organ 79: 43-47.

40. Bartlett JG (2004) Diagnostic test for etiologic agents of community-acquired pneumonia. Infect Dis Clin North Am 18: 809-827.
